# Foraging behavior of Highland cattle in silvopastoral systems in the Alps

**DOI:** 10.1007/s10457-023-00926-z

**Published:** 2023-12-22

**Authors:** Ginevra Nota, Mia Svensk, Davide Barberis, David Frund, Rebecca Pagani, Marco Pittarello, Massimiliano Probo, Simone Ravetto Enri, Michele Lonati, Giampiero Lombardi

**Affiliations:** 1 Department of Agriculture, Forest and Food Sciences, University of Torino, Largo Paolo Braccini 2, 10095 Grugliasco, TO Italy; 2 Agroscope, Grazing Systems, Route de la Tioleyre 4, 1725 Posieux, Switzerland; 3 Ente di Gestione delle Aree Protette delle Alpi Marittime, Piazza Regina Elena 30, 12010 Valdieri, CN Italy; 4 Department of Veterinary Sciences, University of Torino, Largo Paolo Braccini 2, 10095 Grugliasco, TO Italy

**Keywords:** Agroforestry, Diet composition, Feeding preferences, Fodder trees, Hardy breeds, Shrub-encroachment

## Abstract

Trees and shrubs expanded in the last decades in European mountains due to land abandonment and the decrease in grazing pressure, and are expected to further spread also due to climate change. As a consequence of low forage quality and topographic constraints, the management of mountain environments dominated by woody vegetation with livestock is often challenging. Silvopastoral systems based on cattle hardy breeds able to forage on woody plants, such as Highland cattle, could be a suitable option for the management and restoration of such environments. In this study, we used direct observations to explore the foraging behavior of Highland cattle in four study areas across the western Alps. In particular, we assessed: (1) cattle diet composition, (2) the selection for more than 30 tree and shrub species, and (3) the relationships between species consumption and their abundance in the environment. Highland cattle fed on a mixture of both woody and herbaceous species, including between 15 and 46% of woody plants in the diet. Some trees (e.g., *Celtis australis*, *Fraxinus* spp., and *Populus tremula*) and shrubs (e.g., *Frangula alnus*, *Rhamnus* spp., and *Rubus idaeus*) were positively selected by cattle, thus they could be an important forage supplement to their diet. Moreover, the results highlighted that relative species consumption generally increased with increasing species abundance in the environment, suggesting that this cattle breed could be suitable to control shrub expansion in highly encroached areas. The outcomes of this study can support the development of targeted silvopastoral systems in the Alps.

**Supplementary Information:**

The online version contains supplementary material available at 10.1007/s10457-023-00926-z.

## Introduction

In European mountains, trees and shrubs expanded in the last decades to the detriment of open habitats (mainly pasturelands) due to land abandonment and grazing pressure decrease, and are expected to further spread due to the additional effect of climate change (Espunyes et al. 2019). Such changes resulted in a general reduction of the ecosystem services associated to mountain agroecosystems (e.g., forage production, protection from natural hazards, and landscape aesthetic value) (Faccioni et al. 2019; Schirpke et al. 2016) and of plant diversity (Koch et al. 2015; Maurer et al. 2006; Orlandi et al. 2016). Mountain environments dominated by tree and shrub species are scarcely exploited by production-oriented livestock, as they provide lower quality forage than open pastures and are usually located in marginal areas with rough topography. Indeed, woody plants are generally characterized by a higher content of lignin and secondary compounds (e.g., tannins, saponins, alkaloids) than the herbaceous forage (Mahieu et al. 2021; Seidavi et al. 2020). Because of these constraints, many mountain shrub-encroached grasslands, shrublands, and forests are left unmanaged and become dense stands characterized by lower biodiversity (Laiolo et al. 2004; Zehnder et al. 2020).

Silvopastoral systems with hardy cattle breeds could be a suitable option for the management of these environments. Indeed, while production-oriented cattle behave as typical grazers, with grass as their main forage source, hardy cattle breeds can include a greater proportion of woody plants in their diet. For instance, Alberes cattle can feed year-round with a predominantly woody diet in Mediterranean forests of the Pyrenees (Bartolomé et al. 2011). In the Alps, Dexter (Pauler et al. 2022b) and Highland (Pauler et al. 2020; Svensk et al. 2022) cattle are acknowledged to feed on woody species as well. The ability of certain livestock species and breeds to consume woody plants is linked with microbial populations in their rumen able to detoxify secondary metabolites and degrade lignin. This characteristic is well documented in goats (Giger-Reverdin et al. 2020; Silanikove 2000), which are considered as mixed feeders (i.e., they feed on a mixture of both herbaceous and woody species) (Hofmann 1989). In addition to the exploitation of otherwise unused forage resources, livestock grazing can be a valuable ecological restoration tool to contribute in counteracting shrub expansion (Casasús et al. 2007; Öllerer et al. 2019). Moreover, silvopastoral systems can favor a better adaptation of mountain farms to climate change, as woody plants can be an important alternative forage during critical grass shortages (e.g., in summer droughts) and provide shade relief to animals during hot periods (Sales-Baptista and Ferraz-de-Oliveira 2021; Vandermeulen et al. 2018b, 2018a).

Due to their robustness and ability to consume woody plants, Highland cattle were proposed as a tool to control shrub encroachment in different silvopastoral systems and geographic regions, from heterogeneous grass-shrub-woodland communities in The Netherlands (Cromsigt et al. 2018) to coastal dunes in Belgium (Lamoot et al. 2005), North American oak savannas (Harrington and Kathol 2009; Hedtcke et al. 2009), and, more recently, shrub-encroached pastures in the Alps (Pauler et al. 2019; Svensk et al. 2021, 2022). Particularly, in the Alps, Svensk et al. (2022) observed that they could damage *Alnus viridis* (Chaix) DC., which is among the most rapidly expanding shrub species in Central Europe (Anthelme et al. 2007), by the combination of foliage direct consumption, trampling, and mechanical damage to branches. Pauler et al. (2019) observed that grazing by Highland cattle can improve plant diversity in Swiss subalpine pastures and reduce shrub cover more efficiently compared to other cattle breeds.

Despite the ability of Highland cattle to forage on woody plants in the Alps has been recently documented in subalpine pastures by Pauler et al. (2020) and Svensk et al. (2022), no studies explored their foraging behavior across different mountain silvopastoral systems and assessed their feeding preferences for different tree and shrub species. This information would be essential to support the possible development of silvopastoral systems based on the Highland cattle breed in the Alps, for instance by improving the accuracy of carrying capacity calculation in mountain shrub-encroached environments, while addressing their restoration. To fill this knowledge gap, we used direct observations to study the foraging behavior of Highland cattle in four study areas in the western Alps characterized by contrasting woody vegetation. Specifically, the aim of this study was to assess the foraging behavior of Highland cattle in the Alps based on their diet composition, feeding preferences, and the influence of species abundance on plant consumption and selection. We hypothesized that: (1) Highland cattle fed on a mixture of both herbaceous and woody plant species, like in a mixed feeder strategy; (2) some woody plants were positively selected, i.e., they were palatable to Highland cattle; and (3) the relative consumption of plant species was influenced by their abundance in the environment.

## Methods

### Study areas and grazing management

**Fig. 1:**
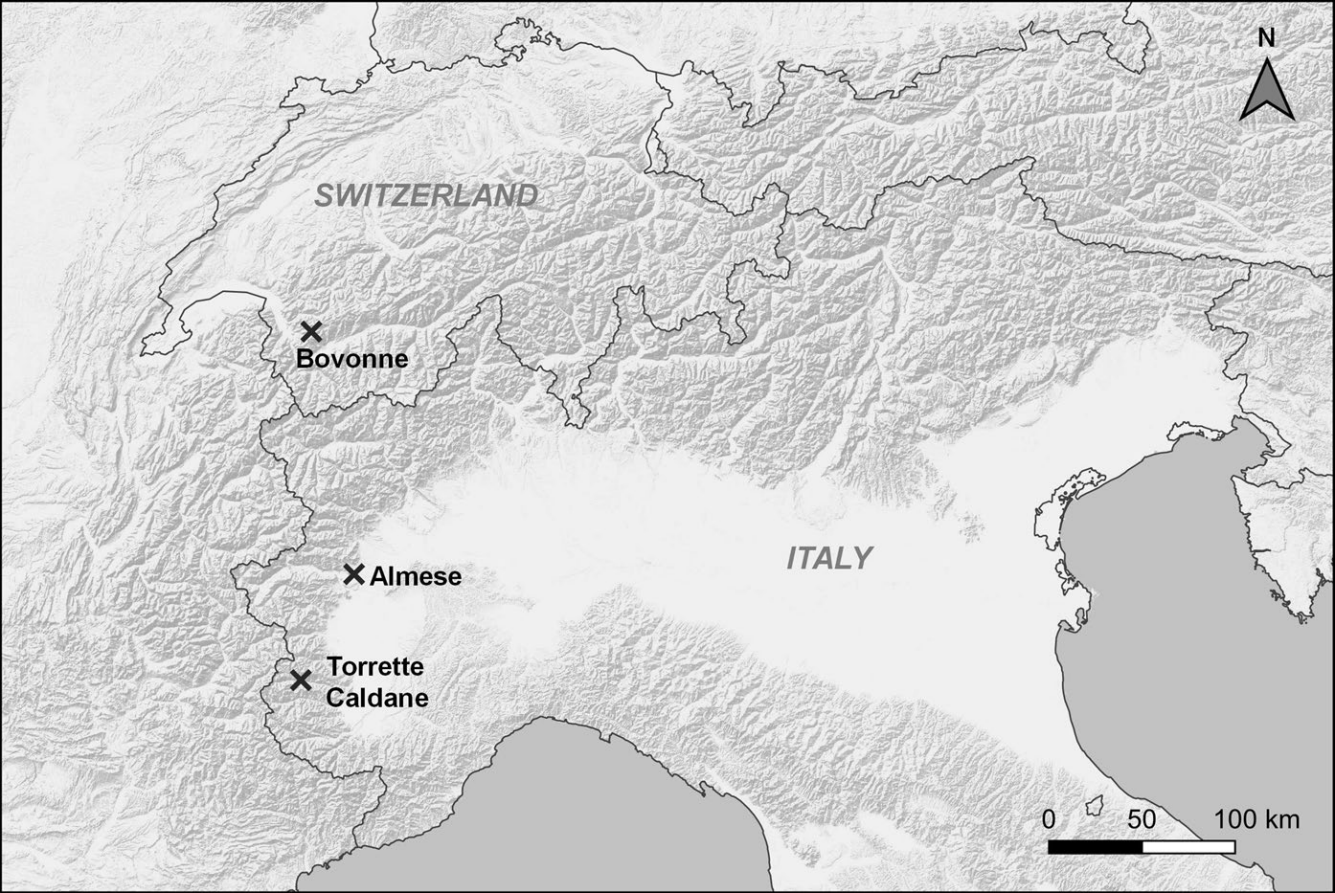
Location of the four study areas (Almese, Torrette, Caldane, and Bovonne) in the western Alps. Basemap: ESRI Terrain

The study was carried out in four paddocks located along an elevation gradient (480–1745 m a.s.l.; Table 1) and extensively grazed by Highland cattle herds in the western Alps, i.e., Almese (Piedmont Region, Italy), Torrette (Piedmont Region, Italy), Caldane (Piedmont Region, Italy), and Bovonne (Canton of Vaud, Switzerland) (Fig. 1). The paddocks were representative of contrasting mountain environments with varying woody plant cover, ranging from 50 to 100% of shrubs and trees (Table 1). In Almese, the vegetation was a mosaic of small meso-xerophile forests, shrublands, and dry-grasslands. Dominant trees were *Fraxinus ornus* L., *Populus tremula* L., and the alien species *Quercus rubra* L.; dominant shrubs were *Prunus spinosa* L. and *Rubus ulmifolius* aggr., while *Bromus erectus* Huds., *Carex caryophyllea* Latourr., and *Chrysopogon gryllus* (L.) Trin. were the most abundant species in the open grassland patches. In Torrette, the vegetation was a deciduous mesophile forest dominated by *Acer pseudoplatanus* L., *Fraxinus excelsior* L., and *Larix decidua* Mill. The shrub *Rubus idaeus* L. was abundant in the understory and dominated the open clearings, while *Festuca flavescens* Bellardi was the most frequent grass. In Caldane, the vegetation was a mosaic of *F. excelsior-* and *Sorbus aria* (L.) Crantz-dominated forests, *P. spinosa* and *Rosa canina* aggr. shrublands, and dry-grasslands dominated by *B. erectus* and *Festuca ovina* aggr. Bovonne was characterized by meso-hygrophile communities dominated by *A. viridis* and by open mesophilous grasslands. In the grassland patches, *Alchemilla xanthochlora* Rothm and *Calamagrostis villosa* (Chaix) J.F.Gmel. were the dominant species, whereas in the *A. viridis* understory, *Adenostyles alliariae* (Gouan) A. Kern. and *Dryopteris dilatata* (Hoffm.) A. Gray were the most abundant species. Aerial photographs and vegetation maps of the four paddocks are available in Online Resource 1.

**Table 1:** Characteristics of the four paddocks of the study (Almese, Torrette, Caldane, and Bovonne)

	Almese	Torrette	Caldane	Bovonne
Coordinates (Datum: WGS84)	45°06′25.2ʺN, 7°26′32.9ʺE	44°34′53.8ʺN 7°05′15.5ʺE	44°35′14.0ʺN, 7°05′39.2ʺE	46°16′09.9ʺN, 7°06′44.3ʺE
Elevation (m a.s.l.)	480	1250	1380	1745
Paddock size (ha)	16.3	14.8	19.0	8.3
Share of woody plant communities (%)	50.4	100.0	56.4	61.0
Livestock Units*	15.4	4.6	12.2	20.7
Herd composition	13 cows, 10 calves, 1 bull	3 cows, 2 heifers, 2 calves	10 cows, 8 calves, 1 bull	9 cows, 9 heifers, 5 calves, 5 young Bulls
Grazing season	late April - mid-June	Mid-August–mid-September	Late June–August	July

*According to EU Regulation 2018/1091 of the European Parliament and of the Council, Annex 1

The study was carried out in the grazing season 2021. The paddocks Almese, Torrette, and Caldane were managed by the same farm. From late April to mid-June, the herd grazed within the Almese paddock. The animals were then moved to summer pastures in the municipality of Casteldelfino, where the herd was divided into two groups: a larger group grazed within the Caldane paddock from late June to the end of August while a smaller group grazed within the Torrette paddock from mid-August to mid-September. Bovonne paddock was grazed for three weeks in July by a different herd. All herds were managed under rotational grazing system based on temporary electric fences. Cows were in the paddocks 24 h per day, free to exploit the available resources without restrictions. Limited amounts of hay were given at the beginning of the grazing period in Almese paddock, due to seasonal drought, which decreased forage availability and quality. Paddock size, livestock units, herd composition, and grazing season are reported in Table 1.

### Direct observations on livestock foraging behavior

To study the foraging behavior, we used direct observations adapting the methodology proposed by Nota et al. (2023) to Highland cattle. Each observer randomly chose an adult cow (focal animal) and recorded its foraging behavior during 15 s. observation sessions followed by 20 s. breaks between each observation. Each cow was continuously observed for two hours, on average, before the observer moved to another focal cow. The identification number of the cow was recorded. We used the feeding station as the spatial scale where decisions on plant selection were made by the cow. According to Bailey et al. (1996) classification, the feeding station is the front feet placement explored by grazing animals during a 5–100 s temporal period. Specifically, we spatially delimited the feeding station as a buffer area having a height of 1.5 m from the ground level and a 50-cm radius around the head of the cow. We assumed that all the plants available in this buffer layer were detectable by the cow and that 1.5-m above ground corresponded to the maximum height that animals could exploit (threshold set according to Svensk et al. 2022, confirmed by our field observations). For each observation session, two variables were recorded: (1) the plant species relative abundance (SA) and (2) the plant species relative consumption (SC). The SA represented the proportion of biomass (ranging from 10 to 100% and visually estimated with a pace of 10%) of each species available in the feeding station. The SC represented the proportion of biomass (ranging from 0 to 100% and visually estimated with a pace of 10%) of each species consumed during the 15 s. session. All woody plant species, tall herbs and ferns were identified at the species level. All other herbaceous species were grouped in a broad category ‘herbage’, as the identification at the species level through direct observations could be extremely difficult for such plants, especially in grassland patches. The nomenclature of plant species followed Aeschimann et al. (2004).

The direct observations were performed during four days in Almese, Torrette, and Caldane, and three days in Bovonne, for a total of 11,286 observation sessions, corresponding to 153 monitoring hours. The monitoring days were performed approximately once every week to be representative of cattle foraging behavior during the whole grazing period in the paddocks.

### Data and statistical analysis

#### Diet composition and Jacobs’ selection index

All analyses were performed separately for the four study areas.

The proportion in the feeding stations (%FS_*i*_) of each woody, tall herb and fern species, and of the ‘herbage’ category was calculated as follows:

$$\%{FS}_{i}= \frac{\sum {SA}_{i}}{{\sum }_{i=1}^{n}{SA}_{i}} \times 100$$

where SA_*i*_ is the abundance of the species *i* or of the ‘herbage’ category at each observation session.

Then, the overall proportion of woody species in the feeding stations was obtained by summing the proportions of all woody plant species. Likewise, the overall proportion of herbaceous species in the feeding stations was obtained by summing the proportions of all tall herbs and ferns, and the ‘herbage’ category.

The proportion in the diet (%DIET_*i*_) of each woody, tall herb and fern species, and of the ‘herbage’ category was calculated as follows:

$$\% DIET_{i} = \frac{{\sum SC_{i} }}{{\mathop \sum \nolimits_{i = 1}^{n} SC_{i} }} \times 100$$

where SC_*i*_ is the consumption of the species *i* or of the ‘herbage’ category at each observation session.

Then, the overall proportion of woody species in the diet was obtained by summing the proportions of all woody plant species. Likewise, the overall proportion of herbaceous species in the diet was obtained by summing the proportions of all tall herbs and ferns, and the ‘herbage’ category.

To assess plant species selection, we calculated the Jacobs’ Selection Index (Jacobs, 1974) according to the following formula:

$$Jacobs^{\prime}\;Selection\;Index_{i} = \frac{{\% DIET_{i} - FS_{i} }}{{\% DIET_{i} + FS_{i} - 2 \times \% DIET_{i} \times FS_{i} }}$$

Jacobs’ Selection Index ranges between − 1 and + 1, with positive values representing preference (i.e., plant species consumed proportionally more than their abundance in the environment), values close to zero representing indifference (i.e., plant species consumed according to their abundance in the environment), and negative values representing avoidance (i.e., plant species consumed proportionally less than their abundance in the environment). The Index was computed only for plant species being recorded in at least 20 observation sessions per paddock and encountered by at least three different cows, to ensure reliability of the results. For the computation of the Index, %FS_*i*_ and %DIET_*i*_ were rescaled to a 0–1 range.

#### Relationships between species consumption and abundance

The relationships between species relative consumption and abundance were scrutinized for two groups of plants: 1) plant species having the upper range of recorded SA values ≥ 80%; and 2) plant species having the upper range of recorded SA values between 50 and 70%. For the first group (= group 1), the data encompassed a large range of SA values, i.e., from sporadic presence to dominance in the feeding station. The second group (= group 2), instead, included species with lower dominance at the feeding station scale.

For both groups, we modeled the SC of plant species as a function of SA through Generalized Additive Models (GAM) assuming a Gaussian distribution for the response variable. Then, for significant GAM curves, we predicted SC values at SA = 10, 20, 30, 40, 50, 60, 70, and 80% for group 1, and SA = 10, 20, 30, 40, and 50% for group 2. For three species which were never consumed by cattle, we could not model their SC because of the absence of variance, thus we attributed zero to all predicted values. To identify different clusters of plant species based on their consumption-abundance relationships, we used the species as response variables and the values predicted with GAMs as explanatory variables to perform a hierarchical cluster analysis (distance matrix: Euclidean; algorithm: Unweighted Pair Group Method with Arithmetic mean, UPGMA). Cluster analyses were performed separately for group 1 and group 2. Finally, for each cluster of species resulting from the cluster analyses, we averaged the predicted values of plant species consumption and plotted the average consumption-abundance relationships.

The analyses were performed with R Software (R Core Team 2018). The ‘mgcv’ package (Wood 2011) was used to fit the GAMs and the ‘vegan’ package (Oksanen et al. 2020) was used to perform the cluster analyses.

## Results

### Diet composition and plant species selection

Thirty different woody plant species were recorded in Almese, 24 in Torrette, 21 in Caldane, and six in Bovonne. About tall herbs and ferns, one species was recorded in Almese, six in Torrette, none in Caldane, and 18 in Bovonne.

**Fig. 2:**
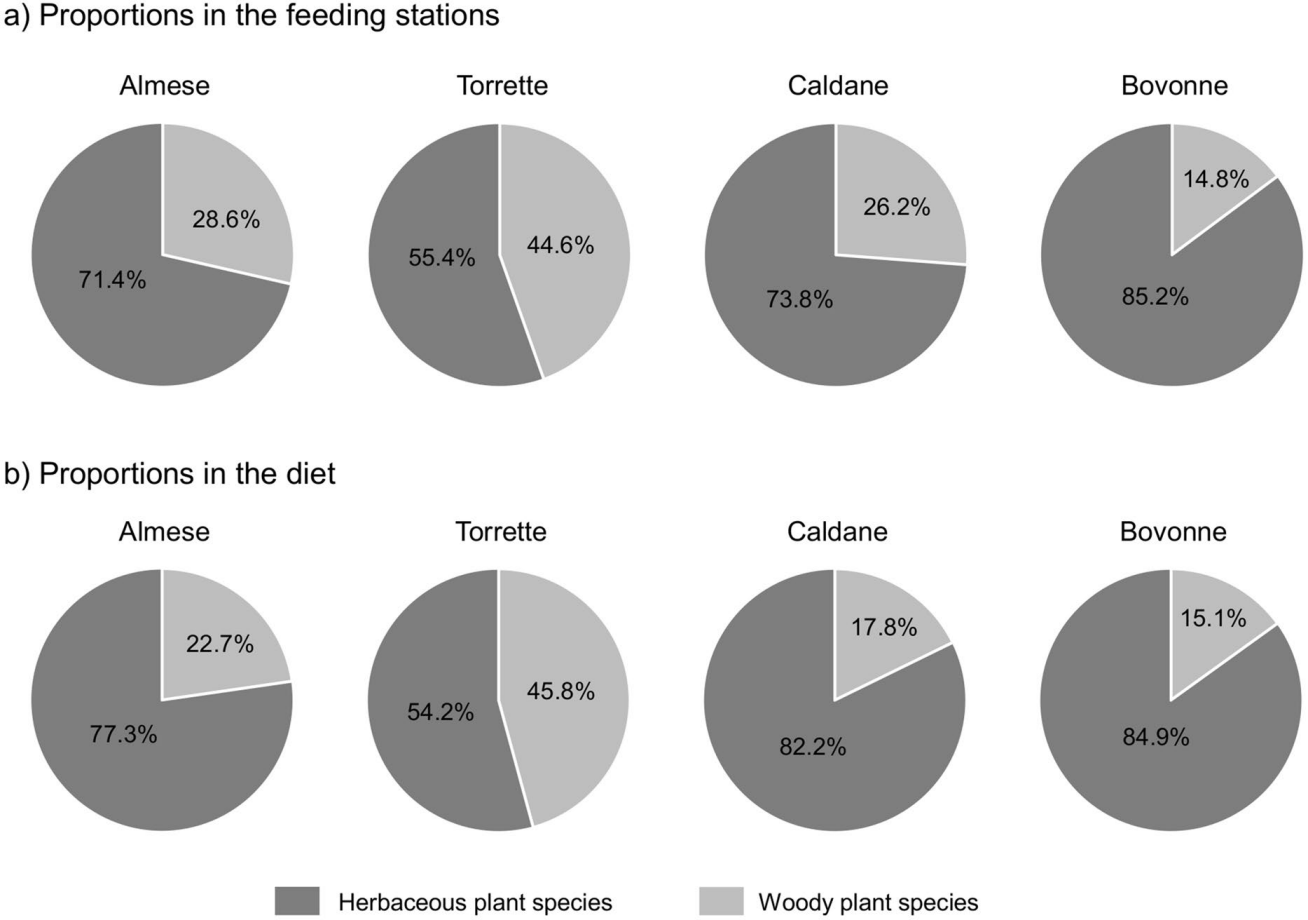
Proportion of woody and herbaceous plant species in **a** the feeding stations and **b** in Highland cattle diet in the four study areas

The proportion of woody species in the feeding stations used by Highland cattle ranged from 14.8% in Bovonne to 44.6% in Torrette (Fig. 2a). The proportions in the diet showed similar patterns to those in the feeding stations, with the lowest proportion of woody species in Bovonne (15.1%), intermediate proportions in Almese and Caldane (28.6% and 26.2%, respectively), and the highest in Torrette (45.8%) (Fig. 2b).

In Almese, the most consumed woody species were *Frangula alnus* Mill. (3.3% of the diet), *P. spinosa* (2.9%), and *F. ornus* (2.9%). *Rubus idaeus* accounted for about 40% of the diet in Torrette, while *P. spinosa* (4.8%) and *R. ulmifolius* aggr. (2.7%) were the main woody plants browsed in Caldane. In Bovonne, *A. viridis* accounted for about 12% of cattle diet. The complete list with all recorded woody and tall herb and fern species, the corresponding number of observations, number of cows that encountered the species, and the proportions in the feeding stations and in cattle diet is reported in the Online Resource 2.

**Fig. 3:**
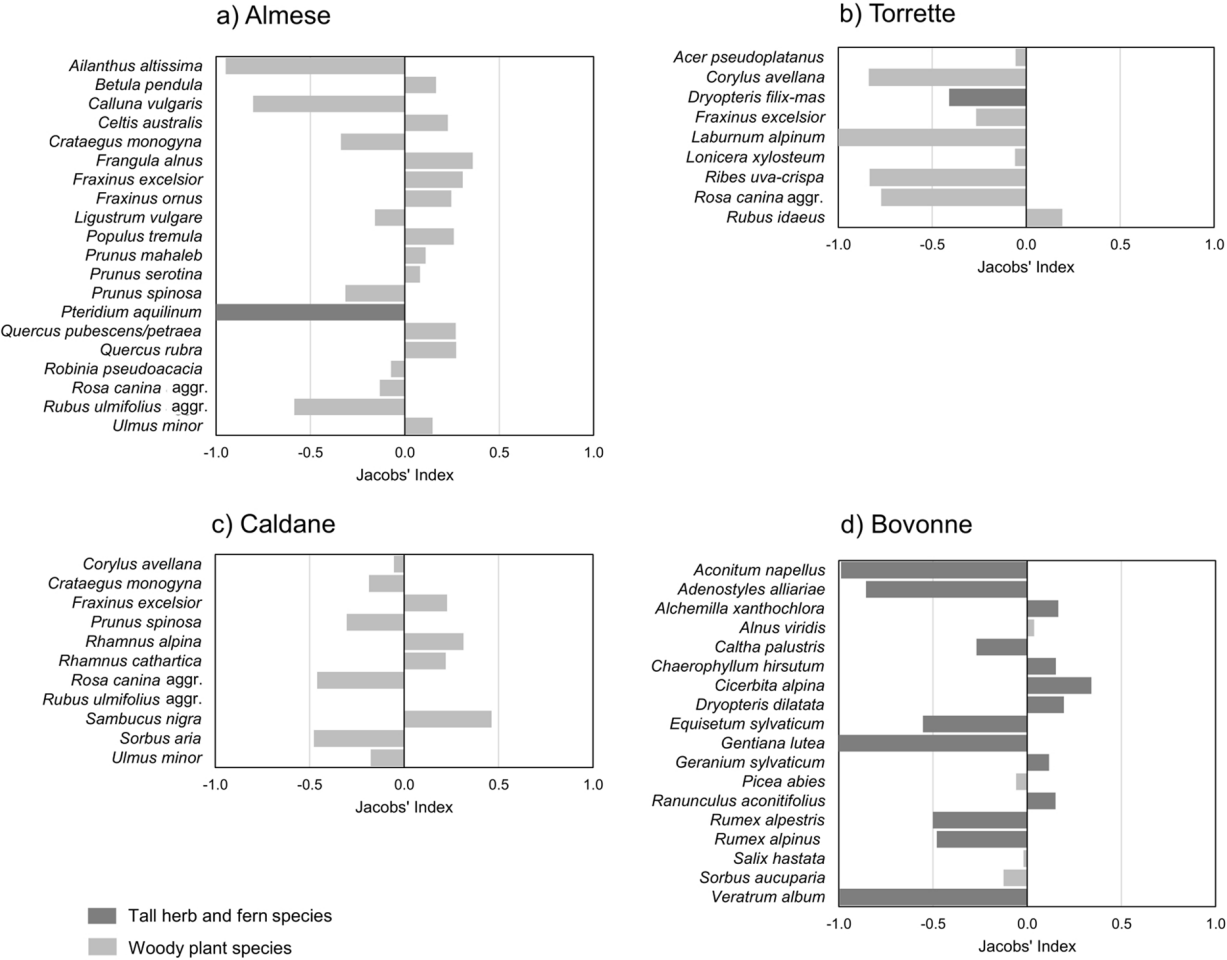
Jacobs’ Selection Index of woody and tall herb and fern species in **a** Almese, **b** Torrette, **c** Caldane, and **d** Bovonne study areas. Positive values indicate preference, values close to zero indicate indifference, negative values indicate avoidance

According to Jacobs’ Selection Index, plant species selection showed a large variability depending on species identity (Fig. 3a–d). Cattle expressed preference for broadleaf trees such as *Celtis australis* L., *P. tremula*, *F. ornus*, and *Quercus* species (i.e., *Q. rubra* and *Q. pubescens/petraea*), and shrubs such as *F. alnus*, *Sambucus nigra* L., *R. idaeus*, and *Rhamnus* species (i.e., *R. alpina* L. and *R. cathartica* L.). Instead, they were rather indifferent (i.e., the relative consumption was equal to their abundance) towards *A. pseudoplatanus*, *A. viridis*, *Picea abies* (L.) H. Karst., and the alien tree *Robinia pseudoacacia* L. Spiny shrubs (i.e., *Crataegus monogyna* Jacq., *P. spinosa*, and *R. canina* aggr.) were moderately avoided, while *Calluna vulgaris* (L.) Hull., *Laburnum alpinum* (Mill.) Bercht. & J. Presl, and the alien tree *Ailanthus altissima* (Mill.) Swingle were strongly refused. For some species, the selection differed depending on the study area, such as for *Corylus avellana* L. (avoided in Torrette and indifferently consumed in Caldane), *F. excelsior* (preferred in Almese and Caldane and avoided in Torrette), *R. ulmifolius* aggr. (avoided in Almese and indifferently consumed in Caldane), and *Ulmus minor* Mill. (preferred in Almese and avoided in Caldane). Among tall herbs, preference was expressed, for instance, towards *Alchemilla xanthochlora* Rothm., *Cicerbita alpina* (L.) Wallr, and *Ranunculus aconitifolius* L., whereas *Aconitum napellus* L., *A. alliariae*, *Gentiana lutea* L., and *Veratrum album* L. were strongly avoided (Fig. 3d). About ferns, *Pteridium aquilinum* (L.) Kuhn and *Dryopteris filix-mas* (L.) Schott were strongly and moderately avoided, respectively, while *D. dilatata* was positively selected.

### Relationships between species consumption and abundance

**Fig. 4:**
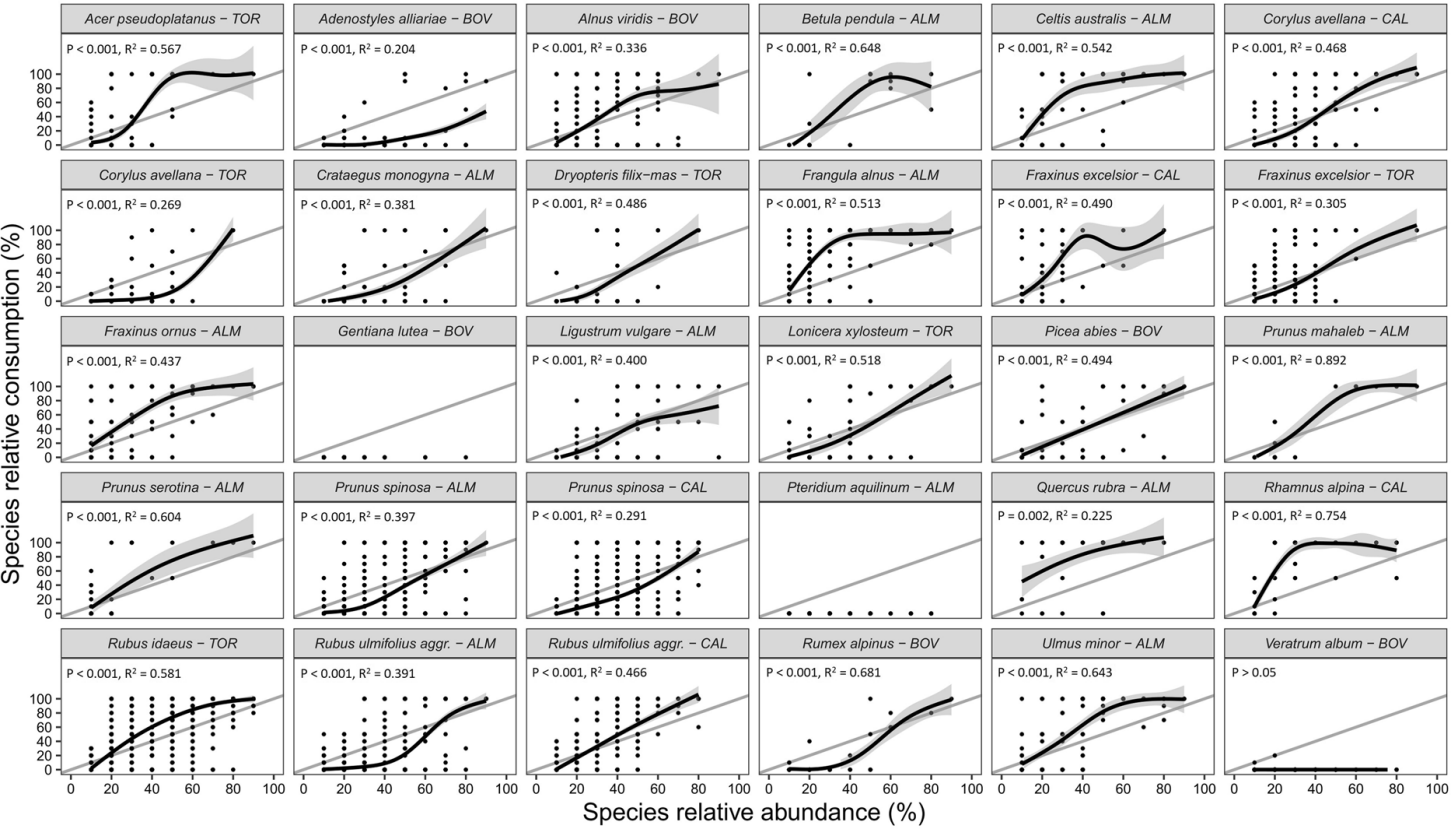
Relationships between species relative consumption and species relative abundance modeled with Generalized Additive Models for group 1 (upper range of species abundance values ≥ 80%) of plant species. *Gentiana lutea* and *Pteridium aquilinum* showed no consumption and thus were not modeled due to the variance equal to 0. R^2^ and p-values are provided in each chart. The gray line is the identity line. Study areas: ALM = Almese, TOR = Torrette, CAL = Caldane, BOV = Bovonne

**Fig. 5:**
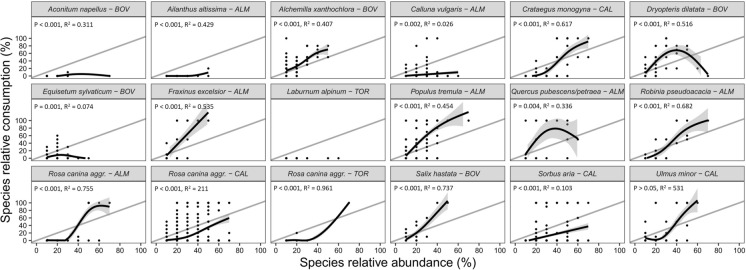
Relationships between species relative consumption and species relative abundance modeled with Generalized Additive Models for group 2 (upper range of species abundance values between 50 and 70%) of plant species. *Laburnum alpinum* showed no consumption and thus was not modeled due to the variance equal to 0. R^2^ and p-values are provided in each chart. The gray line is the identity line. Study areas: ALM = Almese, TOR = Torrette, CAL = Caldane, BOV = Bovonne

Group 1 and group 2 of plant species included 30 and 18 plants, respectively. The relationships between species consumption and abundance resulting from the GAMs for each plant are shown in Figs. 4 and 5. Notably, only one species, *V. album*, had a *p* value > 0.05. Consequently, we opted not to apply a GAM for predicting its SC. Given that *V. album* featured 568 observations with SC = 0, except for one observation with SC = 0.2 (with SA = 0.2) and another with SC = 0.1 (with SA = 0.1), we assumed predicted SC values of 0 for these two species as well (as for *Gentiana lutea* and *Pteridium aquilinum*). For most of other species, the relative consumption increased with increasing abundance in the feeding station, although the shape and slope of these relations differed among plants.

**Fig. 6:**
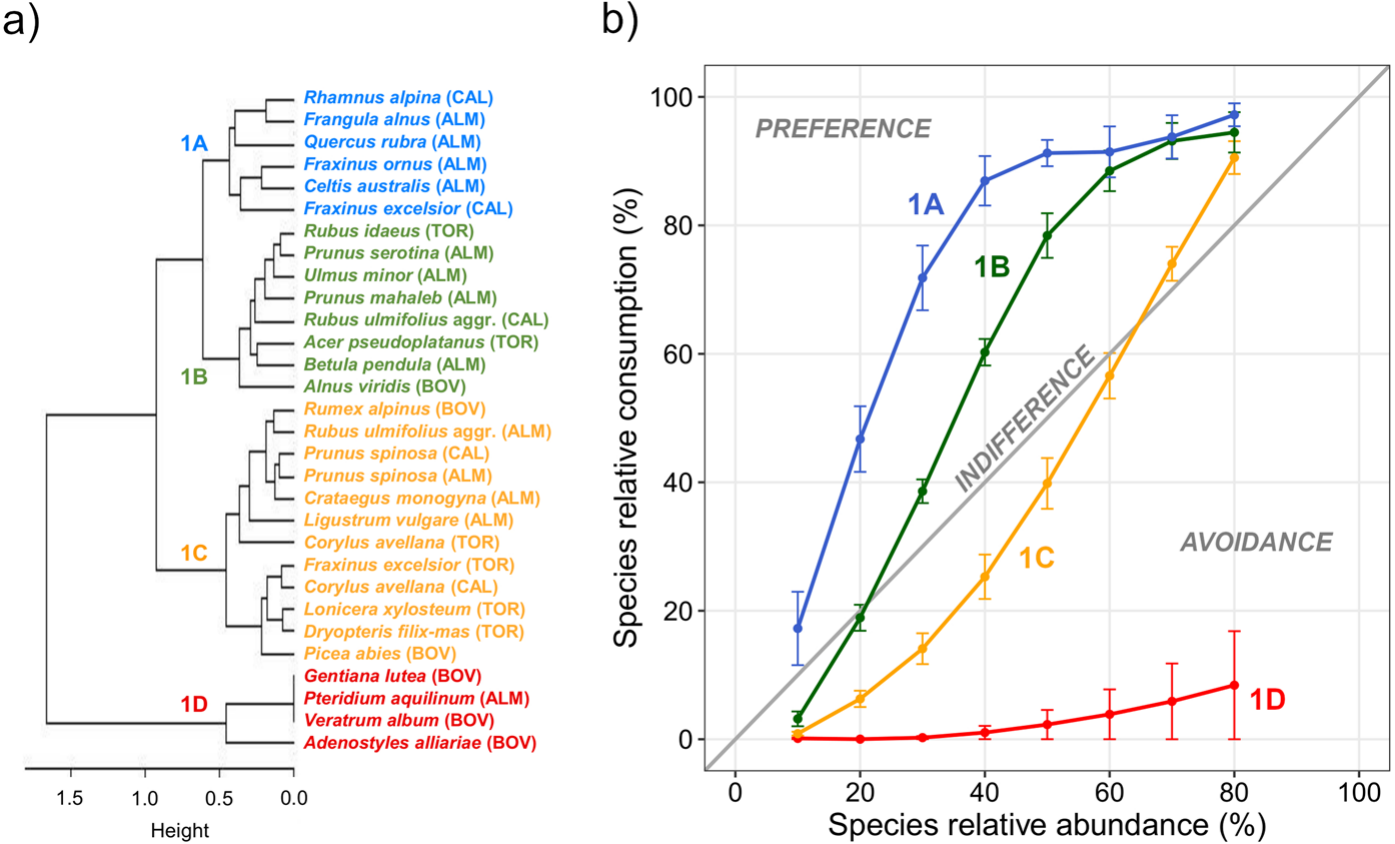
**a** Dendrogram and **b** relationships between species relative consumption and abundance for group 1 (upper range of species abundance values ≥ 80%) of plant species. Different colors highlight different clusters of species (i.e., 1A, 1B, 1C, and 1D). In panel b, values are means and bars are standard errors, and the plot areas above, close to, and below the identity line indicate preference, indifference, and avoidance by cattle, respectively. Study areas: ALM = Almese, TOR = Torrette, CAL = Caldane, BOV = Bovonne

**Fig. 7:**
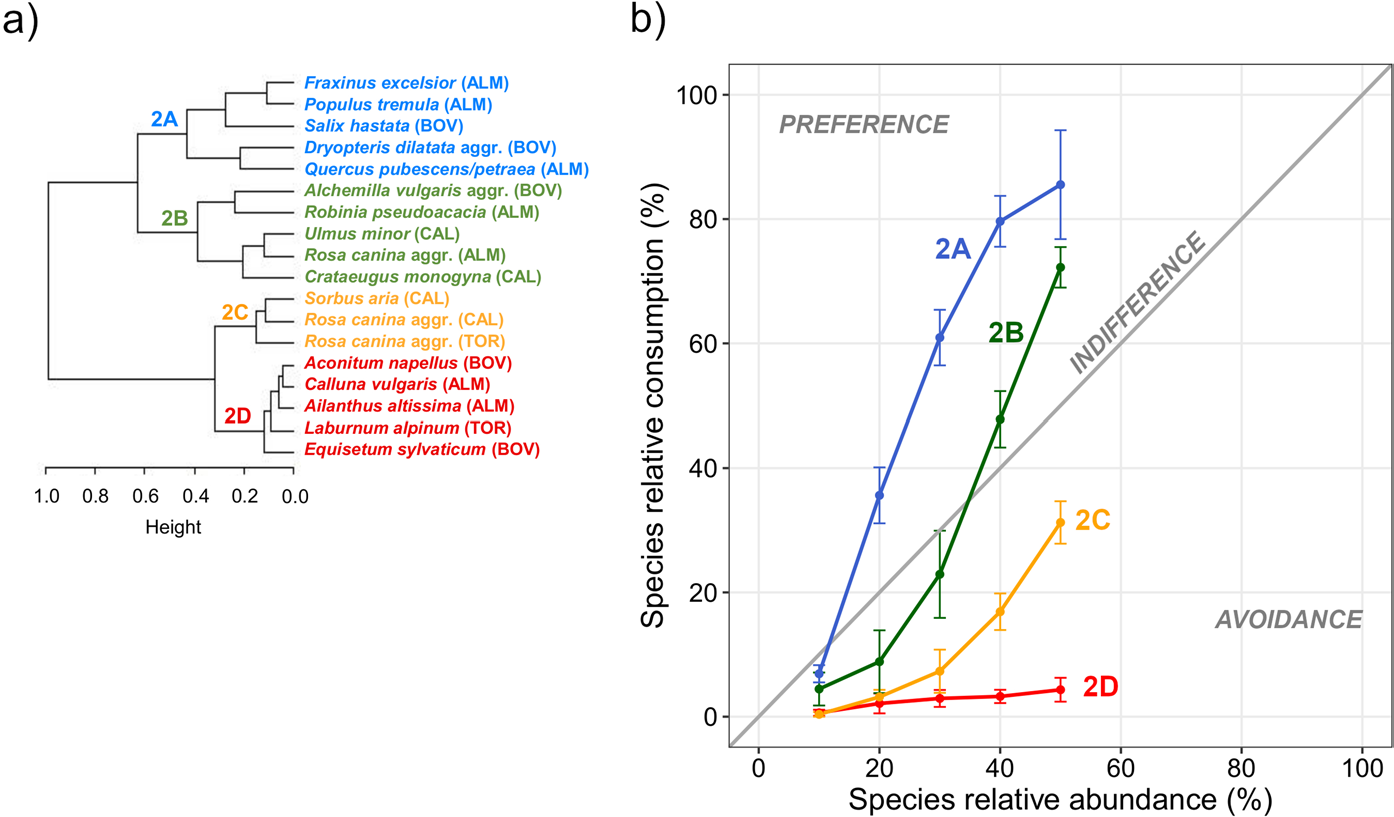
**a** Dendrogram and **b** relations between species relative consumption and abundance for group 2 (upper range of SA values ≥ 50 and ≤ 70%) of plant species. Different colors highlight different clusters of species (i.e., 2A, 2B, 2C, and 2D). In panel b, values are means and bars are standard errors, and the plot areas above, close to, and below the identity line indicate preference, indifference, and avoidance by cattle, respectively. Study areas: ALM = Almese, TOR = Torrette, CAL = Caldane, BOV = Bovonne

The cluster analyses performed with the values predicted with GAMs showed four clusters of species for both group 1 and group 2: 1A, 1B, 1C, and 1D for the first group (Fig. 6a) and 2A, 2B, 2C, and 2D for the second one (Fig. 7a). Each cluster of species was characterized by a distinct relationship between species consumption and abundance and represented a different level of selection by cattle (Figs. 6b and 7b). Particularly, for the plants belonging to clusters 1A (e.g., *F. ornus* and *Q. rubra*) and 2A (e.g., *D. dilatata*), the relationship showed the greatest slope in the first part of the curve compared to all other curves. These species were consumed more than proportionally to their abundance in the feeding station (i.e., they were preferred), also at low SA values. The species belonging to clusters 1B (e.g., *A. viridis* and *R. idaeus*) and 2B (e.g., *R. pseudoacacia*) were consumed less than proportionally to their abundance at low SA values, then more than proportionally when SA were ≥ 20–30%. For the species belonging to clusters 1C (e.g., *C. avellana* and *P. spinosa*) and 2C (i.e., *R. canina* aggr. and *S. aria*), species consumption was less than proportional to their abundance, except at very high SA values (≥ 70%). The species belonging to clusters 1D and 2D showed a very low consumption (< 10%) and were consistently avoided by cattle regardless of their abundance in the feeding station. Among these species, *G. lutea*, *L. alpinum*, and *P. aquilinum* showed no consumption (Fig. 4 and 5). The selection of plant species by cattle as resulting from the consumption-abundance relationships was coherent with Jacobs’ Selection Indices.

## Discussion

This study allowed to explore the foraging behavior of Highland cattle in contrasting Alpine environments characterized by abundant woody vegetation. Based on diet composition, we accept our first hypothesis that Highland cows fed on a mixture of herbaceous and woody plants in all study areas, indicating they behaved as mixed feeders in these environments. Our data of woody plants proportion in cattle diet (15–46%) fall within the range reported by other studies performed with the Highland breed in silvopastoral systems worldwide (14–21% in Lamoot et al. 2005; 21–60% in Hedtcke et al. 2009; around 20% in Cromsigt et al. 2018; around 10% in Pauler et al. 2020). Interestingly, in our study such proportion of woody plants eaten (15–46%) mirrored their proportion in the feeding stations (15–45%) and suggested that cows adapted the diet to the vegetation available in the foraging areas they encountered while grazing. The results also showed that diet composition varied among the different paddocks, likely because of differences in terms of resource availability and forage quality (Bartolomé et al. 2011; Iussig et al. 2015; Mandaluniz et al. 2011; Pauler et al. 2020). For instance, we suggested that cows consumed the largest proportion of trees and shrubs in Torrette because they were very abundant in the paddock (100% of the paddock dominated by woody plants), with *R. idaeus* being a good forage resource (Mahieu et al. 2021), whereas available herbaceous plants (e.g., *F. flavescens*) were less palatable. Contrarily, in Bovonne, trees and shrubs accounted for the lowest proportion of the diet (15%) probably because cows preferred to spend more time in open areas to graze on high quality herbaceous forage (with abundance of e.g., *Festuca nigrescens* and *Phleum rhaeticum*; Svensk et al. (2022) compared to the other sites where grasses were of poorer forage quality (e.g., *F. ovina* aggr. and *B. erectus* in Almese and Caldane).

Results based on Jacobs’ Selection Index and consumption-abundance relationships were coherent and confirmed our second hypothesis that some woody plants were palatable to Highland cattle and could represent an important forage resource in silvopastoral systems. For instance, leaves of *C. australis*, *P. tremula*, and *F. ornus* were positively selected by cows. *Celtis australis* is considered a nutritious and high palatable forage species (Singh et al. 2010), while *P. tremula* is of intermediate quality (Hejcmanová et al. 2014). *Fraxinus ornus* is acknowledged as an important browse species for goats in Mediterranean environments (Papachristou et al. 1999; Papachristou and Papanastasis 1994). *Fraxinus excelsior*, which has high forage quality (Hejcmanová et al. 2014; Ravetto Enri et al. 2020), was positively selected by cows too, except in Torrette study area. The avoidance for this species observed in Torrette may be explained by the age of the plants, as in this paddock there were abundant *F. excelsior* seedlings about 20-cm tall, whereas in the other sites the trees were mainly adult and the cows fed on their lowest branches. The reduction in plant chemical defenses and increase in herbivory with increasing plant age has been documented for some trees (Boege and Marquis 2005). Other plants largely appreciated by cows as browse species were the shrubs *F. alnus*, *S. nigra*, and *R. idaeus*, in line with their high nutritional quality (Mahieu et al. 2021). The leaves of *Rhamnus* species (*R. alpina* and *R. cathartica*) were positively selected too, despite their bark and berries are acknowledged for the presence of toxic compounds (e.g., anthraquinones; Wink 2010). Interestingly, the alien invasive tree *Q. rubra* was palatable to Highland cattle, whereas cows expressed indifference for the alien invasive tree *R. pseudoacacia,* and totally refused the alien invasive tree *A. altissima*, which is rich in secondary compounds (Kowarik and Säumel 2007). Selection of *A. pseudoplatanus* and *C. avellana*, typical species of European temperate forests, ranged from indifference to avoidance. This result agrees with the low forage quality of their leaves (i.e., low digestibility and high phenols concentration) as documented in literature (Mahieu et al. 2021; Papachristou and Papanastasis 1994; Ravetto Enri et al. 2020). As recently observed by Svensk et al. (2022), Highland cattle fed on *A. viridis* leaves, which were consumed according to their abundance in the environment. Despite its moderately high tannin concentration (Stević et al. 2010), this shrub’s foliage is rich in protein (Bühlmann et al. 2016; Pauler et al. 2022a) and could represent an important constituent of cattle diet in subalpine pastures. When foraging in *A. viridis* shrublands, cows expressed a strong selection towards understory species, as they preferred to graze *C. alpina* and the fern *D. dilatata* while completely avoiding other frequent tall herbs such as *A. alliariae* and the toxic *A. napellus* and *V. album*. Additionally, according to its well-known poisonous effects for livestock species (Marrs and Watt 2006), the fern *P. aquilinum* was totally refused in Almese. Finally, cows expressed neutral to negative selection for spiny shrubs such as *C. monogyna*, *P. spinosa*, *R. canina* aggr., and *R. ulmifolius* aggr. Despite the leaf quality of such shrubs is rather high (excellent in *P. spinosa*; Mahieu et al. 2021), spines represented an important deterrent to browsing. Despite these novel and interesting outcomes, the selection for some of the plant species we studied, although commonly distributed in Alpine environments, was based on the data from only one paddock. Paddocks were characterized by different ecological, botanical, management, and seasonal conditions, which affected plant species selection. Consequently, these results should be viewed as a first finding about Highland cattle foraging behavior in the Alps.

The information about cattle feeding preferences obtained with this study could contribute to a better definition of the forage provision and the vegetation carrying capacity in silvopastoral systems managed with Highland cattle. Indeed, in the Alps, the computation of the vegetation carrying capacity has been developed for pastures and is based on the quality indices of herbaceous plants, which represent the main forage source for conventional cattle breeds, whereas trees and shrubs are generally considered with low nutritional value for domestic ruminants. For instance, several indices of specific quality targeted for herbaceous plants (Cavallero et al. 2007; Roggero et al. 2002) have been defined for the calculation of the pastoral value sensu Daget and Poissonet (1971). The inclusion of woody plants in the estimation of the vegetation carrying capacity, such as through the definition of their specific quality indices (see for example the study by Gusmeroli et al. 2007), could help a better and sustainable management of these mountain environments.

Finally, based on consumption-abundance relationships, we accept our third hypothesis that species consumption was influenced by their abundance in the feeding station. Particularly, most of the species were increasingly consumed when they were increasingly available to cows. These results agree with other studies on grass (Agnusdei and Mazzanti 2001; Chen et al. 2015) and woody (Elias and Tischew 2016) species performed at the pasture scale. Exceptions to this trend were the strongly avoided species, i.e., those of clusters 1D and 2D, which were seldom or never eaten regardless of their abundance, probably because of high unpalatability and toxicity issues. Interestingly, consumption-abundance relationships showed that the selection by cattle for some species (i.e., clusters 1B, 2B, and 1C) varied from avoidance to preference along the gradient of increasing species abundance. This trend may be explained in the context of momentary maximization theories, which assume that animals select the best available alternative at any given time (Bailey et al. 1996; Senft et al. 1987). Particularly, the most palatable plants in the feeding station are selected until palatability of remaining forage decreases to a threshold value, then cows move to another feeding station. The threshold value is not fixed but increases when animals encounter high quality plants and decreases with lower quality plants (Bailey et al. 1996; Senft et al. 1987). According to this interpretation, when cows are foraging in a feeding station highly encroached by tree and shrub species of medium–low palatability (i.e., those of clusters 1B, 2B, and 1C), the threshold decreases and the consumption and selection on these plants increase. Conversely, when these species are occasionally present in the feeding station, they are avoided as cows prefer to forage on more palatable species. In this regard, we highlight that Jacobs’ Selection Index was ineffective to detect changes of species selection along the gradient of increasing abundance as it only provides an overall selection value. Consumption-abundance relationships, instead, allowed to scrutinize more thoroughly feeding preferences, highlighting how some generally avoided plants can be positively selected when their abundance is high. This behavior has implications especially for species like *A. viridis*, *P. spinosa*, *R. canina* aggr., and *R. ulmifolius* aggr., whose encroachment into open grasslands represents a crucial issue in European mountains due to land abandonment (Casasús et al. 2007; Verdinelli et al. 2022). Our results highlighted that browsing on these species is more effective when cows are foraging in highly encroached patches rather than when these species are sparsely distributed within a matrix of more palatable species. Silvopastoral systems with the Highland breed may thus represent a management tool to control undesirable woody plant species, particularly when cows exploit highly encroached areas. Increases in the animal stocking rates (Pauler et al. 2022b) and the use of attractive points (Pittarello et al. 2016; Svensk et al. 2022) could further contribute to intensify the impact of cattle on target trees and shrubs.

## Conclusions

This study showed that Highland cattle had a mixed diet consisting of both woody and herbaceous plants, suggesting that silvopastoral systems based on this breed could be a valuable option for the management and restoration of abandoned environments in the Alps. Some trees (e.g., *Celtis australis*, *Fraxinus* spp., and *Populus tremula*) and shrubs (e.g., *Frangula alnus*, *Rhamnus* spp., and *Rubus idaeus*) were very palatable to Highland cattle, thus can be an important forage resource and a supplement to cattle diet. In addition, our results highlighted that cows generally increased the consumption of plant species with their increasing abundance in the feeding station, suggesting that this breed may be suitable to control shrub expansion in highly encroached areas. Further research should integrate foraging behavior evaluations with tree and shrub forage quality and animal performance analyses. Moreover, the effects of the grazing management with Highland cattle on the restoration of shrub-encroached grasslands, shrublands, and forests should be assessed in the long term.

### Supplementary Information

Below is the link to the electronic supplementary material.

Supplementary file1 (DOCX 32 KB)

Supplementary file2 (DOCX 2149 KB)

## Data Availability

Data will be made available on request.

## References

[CR59] Aeschimann D, Lauber K, Moser DM, Theurillat JP (2004) Flora alpina: Atlante delle 4500 piante vascolari delle Alpi. Zanichelli

[CR1] Agnusdei MG, Mazzanti A (2001). Frequency of defoliation of native and naturalized species of the Flooding Pampas (Argentina): defoliation of Flooding Pampas species under grazing. Grass Forage Sci.

[CR2] Anthelme F, Villaret J, Brun J (2007). Shrub encroachment in the Alps gives rise to the convergence of sub-alpine communities on a regional scale. J Veg Sci.

[CR3] Bailey DW, Gross JE, Laca EA, Rittenhouse LR, Coughenour MB, Swift DM, Sims PL (1996). Mechanisms that result in large herbivore grazing distribution patterns. J Range Manag.

[CR4] Bartolomé J, Plaixats J, Piedrafita J, Fina M, Adrobau E, Aixàs A, Bonet M, Grau J, Polo L (2011). Foraging behavior of alberes cattle in a Mediterranean forest ecosystem. Rangel Ecol Manag.

[CR5] Boege K, Marquis RJ (2005). Facing herbivory as you grow up: the ontogeny of resistance in plants. Trends Ecol Evol.

[CR6] Bühlmann T, Körner C, Hiltbrunner E (2016). Shrub expansion of *Alnus viridis* drives former montane grassland into nitrogen saturation. Ecosystems.

[CR7] Casasús I, Bernués A, Sanz A, Villalba D, Riedel JL, Revilla R (2007). Vegetation dynamics in Mediterranean forest pastures as affected by beef cattle grazing. Agric Ecosyst Environ.

[CR8] Cavallero A, Aceto P, Gorlier A, Lombardi G, Lonati M, Martinasso B, Tagliatori C (2007) I tipi pastorali delle Alpi piemontesi. Alberto Perdisa Editore

[CR9] Chen WQ, Wang XY, Zhang YJ, Huang D (2015). Effects of the vertical and horizontal availability of food resources: the diet selection of sheep grazing on natural grassland. J Agric Sci.

[CR10] Cromsigt JPGM, Kemp YJM, Rodriguez E, Kivit H (2018). Rewilding Europe’s large grazer community: how functionally diverse are the diets of European bison, cattle, and horses?. Restor Ecol.

[CR11] Daget P, Poissonet J (1971). Une méthode d’analyse phytologique des prairies. Annales Agronomiques.

[CR12] Elias D, Tischew S (2016). Goat pasturing—a biological solution to counteract shrub encroachment on abandoned dry grasslands in Central Europe?. Agric Ecosyst Environ.

[CR13] Espunyes J, Lurgi M, Büntgen U, Bartolomé J, Calleja JA, Gálvez-Cerón A, Peñuelas J, Claramunt-López B, Serrano E (2019). Different effects of alpine woody plant expansion on domestic and wild ungulates. Glob Change Biol.

[CR14] Faccioni G, Sturaro E, Ramanzin M, Bernués A (2019). Socio-economic valuation of abandonment and intensification of Alpine agroecosystems and associated ecosystem services. Land Use Pol.

[CR15] Giger-Reverdin S, Domange C, Broudiscou LP, Sauvant D, Berthelot V (2020). Rumen function in goats, an example of adaptive capacity. J Dairy Res.

[CR16] Gusmeroli F, Della Marianna G, Puccio C, Corti M, Maggioni L (2007). Indici Foraggeri di specie legnose ed erbacee alpine per il bestiame caprino. Quaderno Sozooalp.

[CR17] Harrington JA, Kathol E (2009). Responses of shrub midstory and herbaceous layers to managed grazing and fire in a North American Savanna (Oak Woodland) and prairie landscape. Restor Ecol.

[CR18] Hedtcke J, Posner J, Rosemeyer M, Albrecht K (2009). Browsing for conservation: springtime forage value of midstory shrubs of degraded oak savannas in southern Wisconsin. Renew Agr Food Syst.

[CR19] Hejcmanová P, Stejskalová M, Hejcman M (2014). Forage quality of leaf-fodder from the main broad-leaved woody species and its possible consequences for the Holocene development of forest vegetation in Central Europe. Veget Hist Archaeobot.

[CR20] Hofmann RR (1989). Evolutionary steps of ecophysiological adaptation and diversification of ruminants: a comparative view of their digestive system. Oecologia.

[CR21] Iussig G, Renna M, Gorlier A, Lonati M, Lussiana C, Battaglini LM, Lombardi G (2015). Browsing ratio, species intake, and milk fatty acid composition of goats foraging on alpine open grassland and grazable forestland. Small Ruminant Res.

[CR60] Jacobs J (1974). Quantitative measurement of food selection. Oecologia.

[CR22] Koch B, Edwards PJ, Blanckenhorn WU, Walter T, Hofer G (2015). Shrub encroachment affects the diversity of plants, butterflies, and grasshoppers on two Swiss subalpine pastures. Arct Antarct Alp Res.

[CR23] Kowarik I, Säumel I (2007). Biological flora of Central Europe: Ailanthus altissima (Mill.) Swingle. Perspect Plant Ecol Evol Syst.

[CR24] Laiolo P, Dondero F, Ciliento E, Rolando A (2004). Consequences of pastoral abandonment for the structure and diversity of the alpine avifauna. J Appl Ecol.

[CR25] Lamoot I, Meert C, Hoffmann M (2005). Habitat use of ponies and cattle foraging together in a coastal dune area. Biol Cons.

[CR26] Mahieu S, Novak S, Barre P, Delagarde R, Niderkorn V, Gastal F, Emile J-C (2021). Diversity in the chemical composition and digestibility of leaves from fifty woody species in temperate areas. Agrofor Syst.

[CR27] Mandaluniz N, Aldezabal A, Oregui LM (2011). Diet selection of beef cattle on Atlantic grassland-heathland mosaic: are heathers more preferred than expected?. Livest Sci.

[CR28] Marrs RH, Watt AS (2006). Biological Flora of the British Isles: Pteridium aquilinum (L.) Kuhn. J Ecol.

[CR29] Maurer K, Weyand A, Fischer M, Stöcklin J (2006). Old cultural traditions, in addition to land use and topography, are shaping plant diversity of grasslands in the Alps. Biol Conserv.

[CR30] Nota G, Berretti R, Ascoli D, Barberis D, Ravetto Enri S, Pittarello M, Motta R, Battaglini LM, Lombardi G, Lonati M (2023). Plant species selection and impact on tree resprouts by semi-free ranging pigs in a temperate deciduous forest. Agrofor Syst.

[CR31] Oksanen J, Guillaume Blanchet F, Friendly M, Kindt R, Legendre P, McGlinn D, Minchin PR, O’Hara RB, Simpson GL, Solymos P, Stevens MHH, Szoecs E, Wagner H (2020). Vegan: community ecology package. R Package Version.

[CR32] Öllerer K, Varga A, Kirby K, Demeter L, Biró M, Bölöni J, Molnár Z (2019). Beyond the obvious impact of domestic livestock grazing on temperate forest vegetation—a global review. Biol Conserv.

[CR33] Orlandi S, Probo M, Sitzia T, Trentanovi G, Garbarino M, Lombardi G, Lonati M (2016). Environmental and land use determinants of grassland patch diversity in the western and eastern Alps under agro-pastoral abandonment. Biodivers Conserv.

[CR34] Papachristou TG, Papanastasis VP (1994). Forage value of Mediterranean deciduous woody fodder species and its implication to management of silvo-pastoral systems for goats. Agrofor Syst.

[CR35] Papachristou TG, Platis PD, Papanastasis VP, Tsiouvaras CN (1999). Use of deciduous woody species as a diet supplement for goats grazing Mediterranean shrublands during the dry season. Anim Feed Sci Technol.

[CR36] Pauler CM, Isselstein J, Braunbeck T, Schneider MK (2019). Influence of Highland and production-oriented cattle breeds on pasture vegetation: a pairwise assessment across broad environmental gradients. Agric Ecosyst Environ.

[CR37] Pauler CM, Isselstein J, Berard J, Braunbeck T, Schneider MK (2020). Grazing allometry: anatomy, movement, and foraging behavior of three cattle breeds of different productivity. Front Vet Sci.

[CR38] Pauler CM, Zehnder T, Staudinger M, Lüscher A, Kreuzer M, Berard J, Schneider MK (2022). Thinning the thickets: foraging of hardy cattle, sheep and goats in green alder shrubs. J Appl Ecol.

[CR39] Pauler CM, Lüscher A, Kreuzer M, Bérard J, Schneider MK, (2022a). Robust cattle, sheep and goats in green alder shrubs – or how to preserve mountain pastures, in: Grassland at the Heart of Circular and Sustainable Food Systems - Proceedings of the 29th General Meeting of the European Grassland Federation, Grassland Science in Europe. L. Delaby, R. Baumont, V. Brocard, S. Lemauviel-Lavenant, S. Plantureux, F. Vertès, J.L. Peyraud, Caen, France, pp 247–249

[CR40] Pittarello M, Probo M, Lonati M, Lombardi G (2016). Restoration of sub-alpine shrub-encroached grasslands through pastoral practices: effects on vegetation structure and botanical composition. Appl Veg Sci.

[CR41] R Core Team, 2018. R: A language and environment for statistical computing. R Foundation for Statistical Computing, Vienna, Austria.

[CR42] Ravetto Enri S, Probo M, Renna M, Caro E, Lussiana C, Battaglini LM, Lombardi G, Lonati M (2020). Temporal variations in leaf traits, chemical composition and in vitro true digestibility of four temperate fodder tree species. Anim Prod Sci.

[CR43] Roggero PP, Bagella S, Farina R (2002). Un archivio dati di Indici specifici per la valutazione integrata del valore pastorale. Rivista Di Agronomia.

[CR44] Sales-Baptista E, Ferraz-de-Oliveira MI (2021). Grazing in silvopastoral systems: multiple solutions for diversified benefits. Agrofor Syst.

[CR45] Schirpke U, Timmermann F, Tappeiner U, Tasser E (2016). Cultural ecosystem services of mountain regions: modelling the aesthetic value. Ecol Indic.

[CR46] Seidavi A, Tavakoli M, Rasouli B, Corazzin M, Salem AZM (2020). Application of some trees/shrubs in ruminant feeding: a review. Agrofor Syst.

[CR47] Senft RL, Coughenour MB, Bailey DW, Rittenhouse LR, Sala OE, Swift DM (1987). Large herbivore foraging and ecological hierarchies. Bioscience.

[CR48] Silanikove N (2000). The physiological basis of adaptation in goats to harsh environments. Small Ruminant Res.

[CR49] Singh B, Bhatt BP, Prasad P (2010). Altitudinal variation in nutritive value of adult-juvenile foliage of *Celtis*
*australis* L. J Am Sci.

[CR50] Stević T, Šavikin K, Zdunić G, Stanojković T, Juranić Z, Janković T, Menković N (2010). Antioxidant, cytotoxic, and antimicrobial activity of *Alnus incana* (L.) ssp. *incana* Moench and *A. viridis* (Chaix) DC ssp. *viridis* extracts. J Med Food.

[CR51] Svensk M, Pittarello M, Nota G, Schneider MK, Allan E, Mariotte P, Probo M (2021). Spatial distribution of highland cattle in *Alnus viridis* encroached subalpine pastures. Front Ecol Evol.

[CR52] Svensk M, Nota G, Mariotte P, Pittarello M, Barberis D, Lonati M, Allan E, Perotti E, Probo M (2022). Use of molasses-based blocks to modify grazing patterns and increase highland cattle impacts on*Alnus viridis*-encroached pastures. Front Ecol Evol.

[CR53] Vandermeulen S, Ramírez-Restrepo CA, Beckers Y, Claessens H, Bindelle J (2018). Agroforestry for ruminants: a review of trees and shrubs as fodder in silvopastoral temperate and tropical production systems. Anim Prod Sci.

[CR54] Vandermeulen S, Ramírez-Restrepo CA, Marche C, Decruyenaere V, Beckers Y, Bindelle J (2018). Behaviour and browse species selectivity of heifers grazing in a temperate silvopastoral system. Agrofor Syst.

[CR55] Verdinelli M, Pittarello M, Caria MC, Piga G, Roggero PP, Marrosu GM, Arrizza S, Fadda ML, Lombardi G, Lonati M, Nota G, Sitzia M, Bagella S (2022). Congruent responses of vascular plant and ant communities to pastoral land-use abandonment in mountain areas throughout different biogeographic regions. Ecol Process.

[CR56] Wink M (2010). Mode of action and toxicology of plant toxins and poisonous plants. Julius-Kühn-Archiv.

[CR57] Wood SN (2011). Fast stable restricted maximum likelihood and marginal likelihood estimation of semiparametric generalized linear models. J R Statis Soc Series B (statis Methodol).

[CR58] Zehnder T, Lüscher A, Ritzmann C, Pauler CM, Berard J, Kreuzer M, Schneider MK (2020). Dominant shrub species are a strong predictor of plant species diversity along subalpine pasture-shrub transects. Alp Bot.

