# Supplementary material for: Foraging behavior of Highland cattle in silvopastoral systems in the Alps
Source: Agrofor Syst. 2023 Dec 22;98(2):491–505. doi: 10.1007/s10457-023-00926-z (PMC10830757; doi:10.1007/s10457-023-00926-z)
Supplement: Supplementary file 1 — Supplementary file1 (DOCX 32 KB) [file 10457_2023_926_MOESM1_ESM.docx]

**Online Resource 2** to the paper ‘Foraging behavior of Highland cattle in silvopastoral systems in the Alps’, *Agroforestry Systems*, Ginevra Nota, Mia Svensk, Davide Barberis, David Frund, Rebecca Pagani, Marco Pittarello, Massimiliano Probo, Simone Ravetto Enri^(^*^)^, Michele Lonati, Giampiero Lombardi.

^(^*^)^ correspondence to simone.ravettoenri@unito.it

**Table 1.** List of the woody (W) and herbaceous (H) plant species recorded in the paddocks of the four study areas (i.e., Almese, Torrette, Caldane, and Bovonne) during the direct observations, with the corresponding number of observation sessions, number of cows that met the species, and the proportion in the feeding stations and in cattle diet.

| **Study area** | **Plant species** | **Life form** | **Observation sessions (n)** | **Cows that met the species during grazing (n)** | **Proportion in the feeding stations (%)** | **Proportion in the diet (%)** |
| --- | --- | --- | --- | --- | --- | --- |
| Almese | *Ailanthus altissima* | W | 42 | 5 | 0.19 | 0.00 |
| Almese | *Alnus glutinosa* | W | 3 | 1 | 0.02 | 0.02 |
| Almese | *Betula pendula* | W | 24 | 7 | 0.24 | 0.33 |
| Almese | *Calluna vulgaris* | W | 342 | 10 | 1.47 | 0.16 |
| Almese | *Cedrus atlantica* | W | 15 | 3 | 0.09 | 0.00 |
| Almese | *Celtis australis* | W | 85 | 6 | 0.88 | 1.39 |
| Almese | *Clematis recta* | W | 19 | 3 | 0.16 | 0.38 |
| Almese | *Crataegus monogyna* | W | 151 | 11 | 1.16 | 0.58 |
| Almese | *Euonymus europaeus* | W | 15 | 3 | 0.07 | 0.23 |
| Almese | *Ficus carica* | W | 6 | 1 | 0.02 | 0.00 |
| Almese | *Frangula alnus* | W | 212 | 9 | 1.61 | 3.35 |
| Almese | *Fraxinus excelsior* | W | 27 | 5 | 0.15 | 0.27 |
| Almese | *Fraxinus ornus* | W | 211 | 10 | 1.76 | 2.87 |
| Almese | *Hedera helix* | W | 7 | 2 | 0.02 | 0.00 |
| Almese | *Juniperus communis* | W | 18 | 4 | 0.14 | 0.00 |
| Almese | *Ligustrum vulgare* | W | 121 | 8 | 1.07 | 0.78 |
| Almese | *Malus* sp. | W | 12 | 2 | 0.10 | 0.19 |
| Almese | *Populus tremula* | W | 211 | 9 | 1.06 | 1.79 |
| Almese | *Prunus avium* | W | 6 | 4 | 0.03 | 0.10 |
| Almese | *Prunus mahaleb* | W | 25 | 3 | 0.34 | 0.42 |
| Almese | *Prunus serotina* | W | 39 | 7 | 0.29 | 0.35 |
| Almese | *Prunus spinosa* | W | 764 | 11 | 5.38 | 2.87 |
| Almese | *Pteridium aquilinum* | H | 161 | 8 | 1.07 | 0.00 |
| Almese | *Pyrus pyraster* | W | 11 | 2 | 0.08 | 0.18 |
| Almese | *Quercus pubescens/petraea* | W | 30 | 8 | 0.18 | 0.31 |
| Almese | *Quercus rubra* | W | 49 | 7 | 0.65 | 1.12 |
| Almese | *Robinia pseudoacacia* | W | 36 | 4 | 0.20 | 0.17 |
| Almese | *Rosa canina* aggr. | W | 63 | 9 | 0.45 | 0.35 |
| Almese | *Rubus ulmifolius* aggr. | W | 1415 | 12 | 9.22 | 2.59 |
| Almese | *Sorbus aria* | W | 3 | 1 | 0.04 | 0.05 |
| Almese | *Ulmus minor* | W | 138 | 10 | 1.39 | 1.86 |
| Torrette | *Acer pseudoplatanus* | W | 244 | 3 | 2.05 | 1.83 |
| Torrette | *Alnus incana* | W | 4 | 2 | 0.06 | 0.00 |
| Torrette | *Athyrium filix-femina* | H | 27 | 2 | 0.26 | 0.00 |
| Torrette | *Betula pendula* | W | 3 | 1 | 0.02 | 0.00 |
| Torrette | *Corylus avellana* | W | 489 | 3 | 4.37 | 0.40 |
| Torrette | *Dryopteris affinis* | H | 6 | 1 | 0.03 | 0.00 |
| Torrette | *Dryopteris dilatata* | H | 3 | 1 | 0.01 | 0.00 |
| Torrette | *Dryopteris filix-mas* | H | 131 | 3 | 1.09 | 0.46 |
| Torrette | *Fagus sylvatica* | W | 26 | 2 | 0.24 | 0.00 |
| Torrette | *Fraxinus excelsior* | W | 466 | 3 | 3.49 | 2.05 |
| Torrette | *Gymnocarpium dryopteris* | H | 2 | 1 | 0.02 | 0.00 |
| Torrette | *Laburnum alpinum* | W | 55 | 3 | 0.35 | 0.00 |
| Torrette | *Larix decidua* | W | 22 | 2 | 0.15 | 0.00 |
| Torrette | *Lonicera alpigena* | W | 1 | 1 | 0.04 | 0.04 |
| Torrette | *Lonicera xylosteum* | W | 95 | 3 | 1.64 | 1.46 |
| Torrette | *Polystichum aculeatum* | H | 2 | 2 | 0.01 | 0.00 |
| Torrette | *Populus alba* | W | 12 | 2 | 0.08 | 0.06 |
| Torrette | *Prunus avium* | W | 21 | 2 | 0.10 | 0.03 |
| Torrette | *Quercus petraea* | W | 12 | 1 | 0.08 | 0.00 |
| Torrette | *Ribes uva-crispa* | W | 26 | 3 | 0.15 | 0.01 |
| Torrette | *Rosa canina* aggr. | W | 63 | 3 | 0.41 | 0.05 |
| Torrette | *Rubus idaeus* | W | 1354 | 3 | 30.70 | 39.54 |
| Torrette | *Rubus ulmifolius* aggr. | W | 1 | 1 | 0.01 | 0.01 |
| Torrette | *Salix alba* | W | 4 | 2 | 0.04 | 0.04 |
| Torrette | *Salix caprea* | W | 8 | 2 | 0.05 | 0.00 |
| Torrette | *Salix purpurea* | W | 1 | 1 | 0.00 | 0.00 |
| Torrette | *Sorbus aria* | W | 59 | 2 | 0.40 | 0.07 |
| Torrette | *Sorbus aucuparia* | W | 15 | 2 | 0.08 | 0.02 |
| Torrette | *Tilia platyphyllos* | W | 5 | 2 | 0.06 | 0.15 |
| Torrette | *Ulmus minor* | W | 2 | 2 | 0.01 | 0.00 |
| Caldane | *Acer pseudoplatanus* | W | 19 | 3 | 0.63 | 0.72 |
| Caldane | *Berberis vulgaris* | W | 2 | 2 | 0.02 | 0.00 |
| Caldane | *Corylus avellana* | W | 308 | 8 | 2.63 | 2.37 |
| Caldane | *Crataegus monogyna* | W | 88 | 9 | 0.64 | 0.44 |
| Caldane | *Fagus sylvatica* | W | 1 | 1 | 0.00 | 0.00 |
| Caldane | *Fraxinus excelsior* | W | 90 | 7 | 0.53 | 0.85 |
| Caldane | *Juniperus communis* | W | 11 | 7 | 0.06 | 0.03 |
| Caldane | *Laburnum alpinum* | W | 3 | 1 | 0.02 | 0.00 |
| Caldane | *Malus domestica* | W | 1 | 1 | 0.01 | 0.03 |
| Caldane | *Prunus avium* | W | 13 | 3 | 0.05 | 0.05 |
| Caldane | *Prunus spinosa* | W | 1096 | 9 | 8.57 | 4.77 |
| Caldane | *Quercus pubescens/petraea* | W | 15 | 5 | 0.05 | 0.00 |
| Caldane | *Rhamnus alpina* | W | 65 | 8 | 0.73 | 1.40 |
| Caldane | *Rhamnus cathartica* | W | 29 | 4 | 0.16 | 0.25 |
| Caldane | *Ribes uva-crispa* | W | 4 | 3 | 0.02 | 0.00 |
| Caldane | *Rosa canina* aggr. | W | 965 | 9 | 5.98 | 2.29 |
| Caldane | *Rubus ulmifolius* aggr. | W | 406 | 9 | 2.72 | 2.72 |
| Caldane | *Salix caprea* | W | 3 | 1 | 0.03 | 0.05 |
| Caldane | *Sambucus nigra* | W | 28 | 5 | 0.17 | 0.46 |
| Caldane | *Sorbus aria* | W | 360 | 9 | 2.62 | 0.94 |
| Caldane | *Ulmus minor* | W | 92 | 6 | 0.55 | 0.39 |
| Bovonne | *Acer pseudoplatanus* | W | 18 | 9 | 0.18 | 0.14 |
| Bovonne | *Aconitum napellus* | H | 126 | 11 | 1.52 | 0.01 |
| Bovonne | *Adenostyles alliariae* | H | 515 | 13 | 11.42 | 0.99 |
| Bovonne | *Alchemilla xanthochlora* | H | 286 | 10 | 4.42 | 6.06 |
| Bovonne | *Alnus viridis* | W | 490 | 14 | 11.10 | 11.85 |
| Bovonne | *Caltha palustris* | H | 40 | 8 | 0.48 | 0.28 |
| Bovonne | *Chaerophyllum hirsutum* | H | 120 | 11 | 1.29 | 1.75 |
| Bovonne | *Cicerbita alpina* | H | 28 | 4 | 0.34 | 0.70 |
| Bovonne | *Cirsium oleraceum* | H | 10 | 4 | 0.09 | 0.16 |
| Bovonne | *Dryopteris dilatata* | H | 52 | 3 | 1.15 | 1.70 |
| Bovonne | *Equisetum sylvaticum* | H | 195 | 12 | 2.35 | 0.69 |
| Bovonne | *Gentiana lutea* | H | 61 | 8 | 1.13 | 0.00 |
| Bovonne | *Geranium sylvaticum* | H | 126 | 11 | 1.48 | 1.86 |
| Bovonne | *Heracleum sphondylium* | H | 10 | 2 | 0.12 | 0.22 |
| Bovonne | *Petasites albus* | H | 1 | 1 | 0.02 | 0.00 |
| Bovonne | *Peucedanum ostruthium* | H | 3 | 2 | 0.04 | 0.03 |
| Bovonne | *Picea abies* | W | 94 | 8 | 2.57 | 2.29 |
| Bovonne | *Ranunculus aconitifolius* | H | 148 | 10 | 1.72 | 2.31 |
| Bovonne | *Rubus idaeus* | W | 6 | 4 | 0.06 | 0.00 |
| Bovonne | *Rumex alpestris* | H | 33 | 6 | 0.33 | 0.11 |
| Bovonne | *Rumex alpinus* | H | 65 | 8 | 1.44 | 0.51 |
| Bovonne | *Salix hastata* | W | 32 | 6 | 0.48 | 0.46 |
| Bovonne | *Sorbus aucuparia* | W | 37 | 9 | 0.42 | 0.32 |
| Bovonne | *Veratrum album* | H | 570 | 14 | 10.16 | 0.03 |
