# Supplementary material for: Foraging behavior of Highland cattle in silvopastoral systems in the Alps
Source: Agrofor Syst. 2023 Dec 22;98(2):491–505. doi: 10.1007/s10457-023-00926-z (PMC10830757; doi:10.1007/s10457-023-00926-z)
Supplement: Supplementary file 2 — Supplementary file2 (DOCX 2149 KB) [file 10457_2023_926_MOESM2_ESM.docx]

**Online Resource 1** to the paper ‘Foraging behavior of Highland cattle in silvopastoral systems in the Alps’, *Agroforestry Systems*, Ginevra Nota, Mia Svensk, Davide Barberis, David Frund, Rebecca Pagani, Marco Pittarello, Massimiliano Probo, Simone Ravetto Enri^(^*^)^, Michele Lonati, Giampiero Lombardi.

^(^*^)^ correspondence to simone.ravettoenri@unito.it


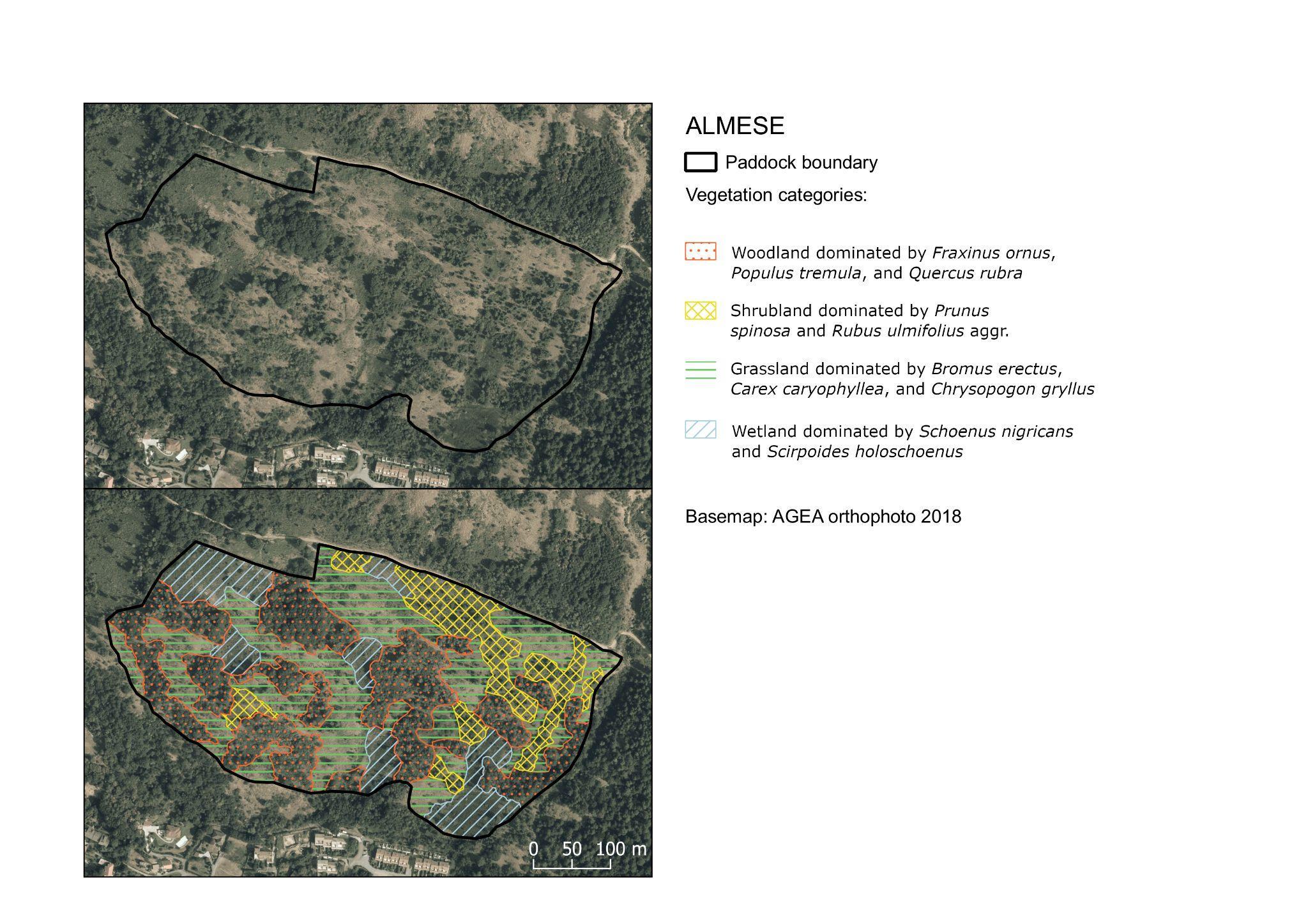


**Figure 1**. Aerial photograph and vegetation map of Almese study area


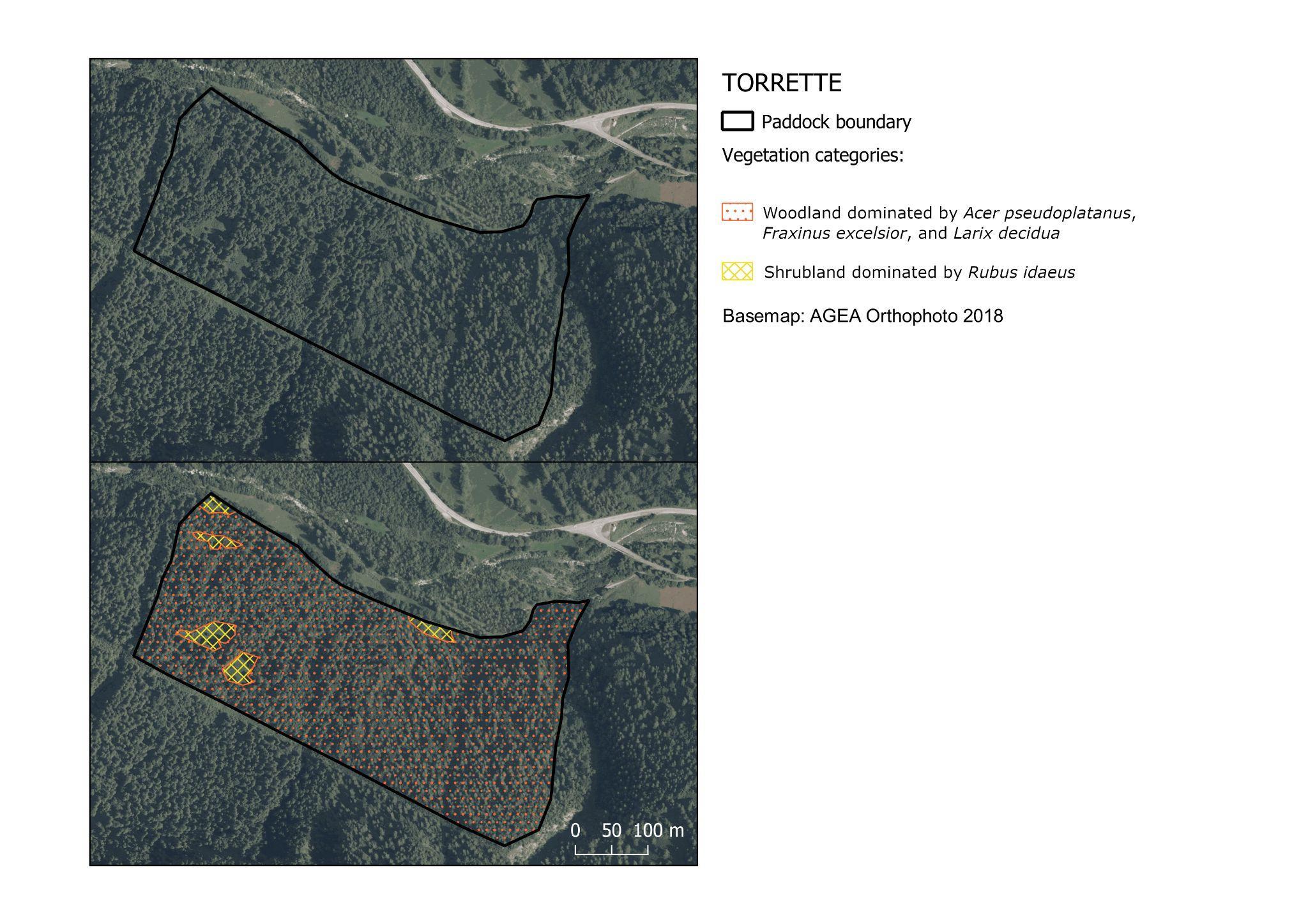


**Figure 2**. Aerial photograph and vegetation map of Torrette study area


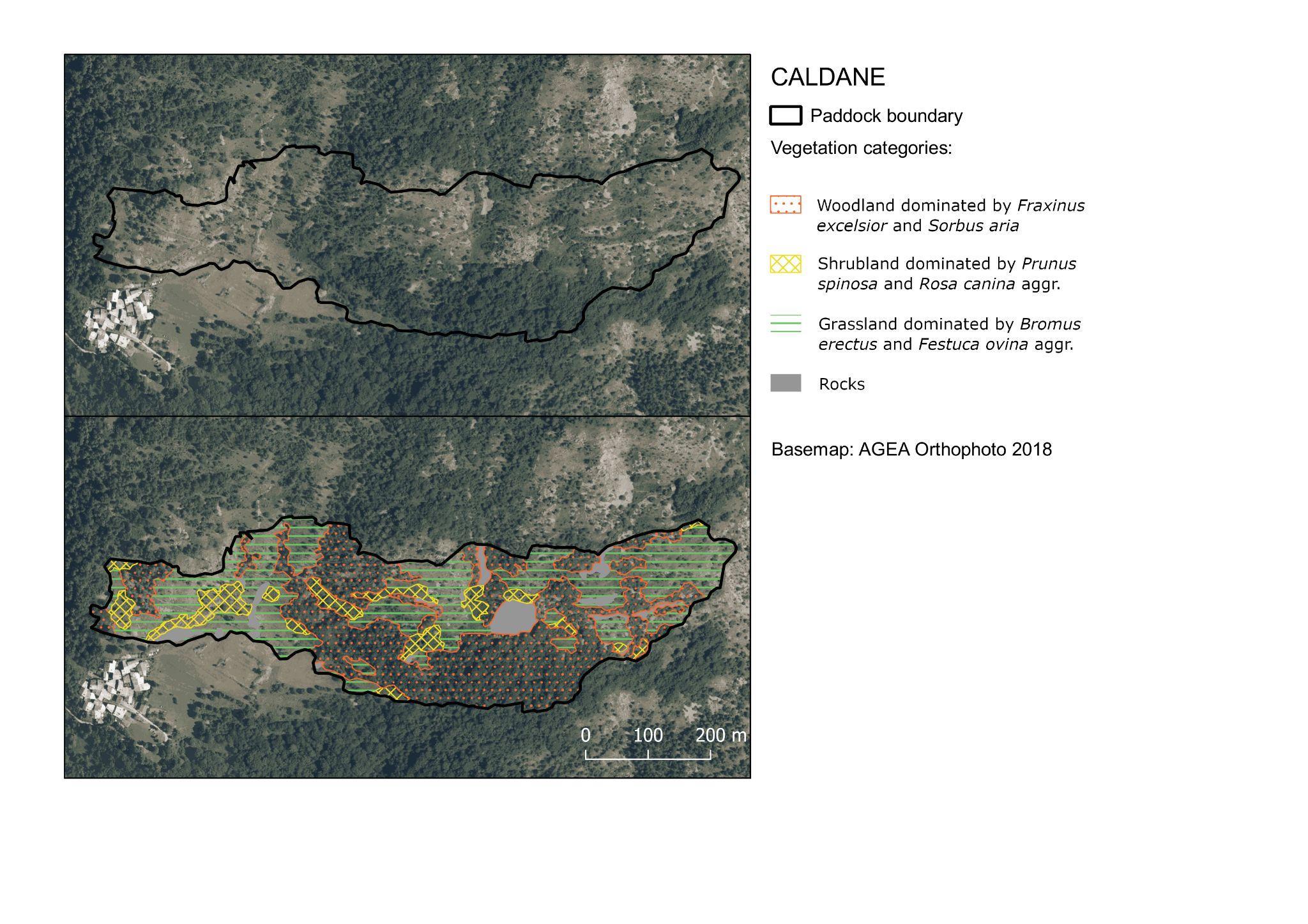


**Figure 3**. Aerial photograph and vegetation map of Caldane study area


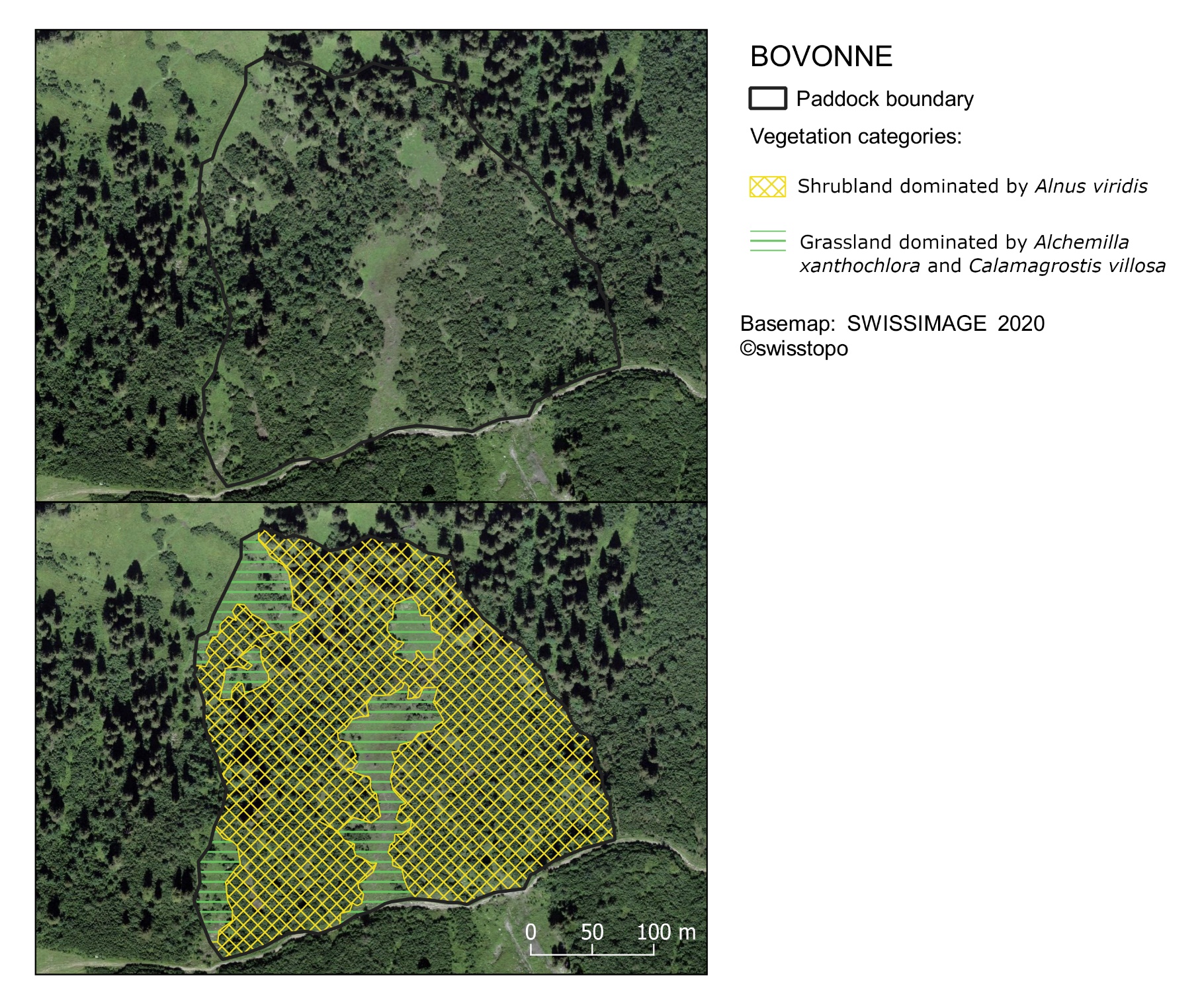


**Figure 4**. Aerial photograph and vegetation map of Bovonne study area
